# The acceptability and effectiveness of eHealth interventions to support assessment and decision-making for people with dementia living in care homes: A systematic review

**DOI:** 10.3389/frdem.2022.977561

**Published:** 2022-09-13

**Authors:** India Tunnard, Juliet Gillam, Catherine Harvey, Nathan Davies, Victoria Vickerstaff, Clare Ellis-Smith, Catherine J. Evans

**Affiliations:** ^1^Department of Palliative Care, Policy and Rehabilitation, Cicely Saunders Institute, King's College London, London, United Kingdom; ^2^Research Department of Primary Care and Population Health, University College London, London, United Kingdom

**Keywords:** dementia, long-term care, systematic review, telemedicine, remote consultation

## Abstract

**Introduction:**

As dementia progresses, care needs increase leading many to require 24-h care in care homes. eHealth interventions have the potential to improve care processes of assessment and decision-making for people with dementia. However, little is known on the acceptability and effectiveness in care homes.

**Aim:**

To identify and explore the components, acceptability and effectiveness of eHealth interventions for people with dementia, families and staff to support assessment and decision-making in care homes.

**Methods:**

A mixed methods systematic review using narrative synthesis. Four databases were searched (Embase, PsycINFO, MEDLINE, and CINAHL) from 2000 to July 2021. Quality appraisal used validated assessment tools appropriate for the study design.

**Results:**

Twenty-six studies met eligibility criteria. Study designs and interventions were heterogeneous. Overall quality was high to moderate. Interventions that promoted supportive, practical learning through integrated working and provided staff with language to communicate resident symptoms were favored by staff. We found evidence that indicated residents were willing to use video consultations; however, families preferred face-to-face consultations. Fifteen studies considered effectiveness. Use of eHealth interventions indicates an improvement in resident outcomes in appropriate prescribing and advance care planning. Staff knowledge, confidence, and wellbeing were also improved. Hospitalisations were reduced when a video consultation component was implemented.

**Discussion:**

Care home staff require support to meet the often multiple and changing care needs of residents with dementia. eHealth interventions can improve outcomes for staff and residents and facilitate integrated working with external professionals to support assessment and management of care. Further work is required to understand acceptability for residents and their families and effectiveness on family outcomes, particularly in non-Western cultures and low-middle income countries.

**Systematic review registration:**

https://www.crd.york.ac.uk/prospero/display_record.php?RecordID=254967, identifier: CRD42021254967.

## Introduction

Dementia is a progressive and terminal syndrome. It is the leading cause of death in the UK (Office of National Statistics, 2020) and globally (Feigin et al., 2019). By 2040, the number of people living with dementia in the UK is projected to increase by over 80% (Wittenberg et al., 2019) and a global increase of 185% by 2050 (Prince et al., 2015). Dementia is characterized by a deterioration in cognitive function, and wider brain functions, which presents as multiple complex care needs that often requires 24-h personalized care until the end of life. This care may be provided by a care home. It is estimated that 70% of care home residents in England are living with dementia, with the average life expectancy on admission to a care home of 1–2 years (British Geriatrics Society, 2020). In total, 58% of all deaths from dementia take place in care homes (Public Health England, 2016).

Assessment and management of care needs for people with dementia can often be challenging due to deteriorating verbal communication. This can cause under detection of distressing symptoms and concerns, leading to unmet needs, increasing distress and compromised quality of life (Corbett et al., 2012). Care home staffs' intrinsic familiarity with their residents means they are well-positioned to assess and identify changes in needs and requirements for care by working with external healthcare providers, such as specialist dementia or palliative care (Hendrix et al., 2003; Ellis-Smith et al., 2017).

The eHealth interventions can facilitate integration with external healthcare professionals by providing remote access to clinical expertise and assessment, and monitoring systems. eHealth is defined as “health services and information delivered or enhanced through the internet and related technologies” (Eysenbach, 2001). eHealth interventions vary widely from an electronic tablet used to video call an external professional to an electronic record to a system that collates multiple data sources to create a visualization. They have been demonstrated to support assessment and management of needs in care homes (Gillespie et al., 2019) and can be used in the care home alone, or to report assessments to external services, such as the General Practitioner (GP). eHealth interventions have been shown to improve resident outcomes, particularly in reducing hospitalisations (Gillespie et al., 2019), an outcome associated with more risk for people living with dementia (Shepherd et al., 2019). Due to the COVID-19 pandemic and subsequent restrictions around visiting in care homes, the use of eHealth interventions has increased rapidly, and recent evidence suggests that this is likely to remain once all pandemic restrictions have been eased (Warmoth et al., 2022). Therefore, it is important to understand how eHealth might impact the lives of residents, families, and staff. Currently, little is known about which components of eHealth interventions are acceptable to residents living with dementia, their families and staff and which are effective at improving outcomes. This review aimed to (1) identify the components, (2) explore the acceptability to residents with dementia, their family, and staff, and (3) consider the effectiveness of eHealth interventions to support assessment and decision-making for people living with dementia in care homes.

## Methods

A mixed methods systematic review using narrative synthesis was conducted following Popay's et al. (2006) guidance (Popay et al., 2006) to identify components, explore acceptability, and consider the effectiveness of eHealth interventions to support assessment and decision-making for those living with dementia in care homes. The review followed the Preferred Reporting Items for Systematic Reviews and Meta-Analyses (PRISMA) (Supplementary material 1. PRISMA Checklist). The protocol was registered on PROSPERO (CRD42021254967).

### Search strategy

The following four databases Embase, PsycINFO, MEDLINE and CINAHL were searched for studies published in English from January 2000 to July 2021. A scoping review of the literature and an Information Support specialist supported the development of the search strategy. Medical Subject Headings (MeSH) terms included dementia AND care homes AND eHealth AND assessment OR decision-making (Supplementary material 2. Search Strategy). Reference chaining and citation tracking were also used to complement the search strategy.

### Eligibility criteria

Participants: Residents of a long-term care facility with a diagnosis of dementia, including within a mixed participant population. Short-term care facilities were excluded.

Intervention: eHealth interventions to support comprehensive assessment of residents and/or to improve decision-making about care and treatment. Non-digitized interventions were out of the scope of this review.

Outcome: All outcome measures relating to acceptability and effectiveness of eHealth interventions used to improve assessment and decision-making on care and treatment in care homes.

Comparator: No restrictions.

Study design: All study designs that report acceptability and effectiveness outcomes relating to assessment and decision-making surrounding care and treatment were eligible for inclusion. Non-English language studies, opinion pieces, editorials, and PhD theses were excluded.

### Study selection

Identified studies were managed using the EndNote X9 reference management system. Two reviewers screened all titles and abstracts (IT and JG) with a review of a random 20% of articles by another blind reviewer (EY, JA, and CH) to assess the rigor of the eligibility criteria by reviewing consistency between reviewers. Two reviewers (IT and JG) considered all full-text articles for eligibility and discussed any uncertainty encountered. Uncertainty that could not be resolved was discussed with the wider research team.

### Quality appraisal

Quality was appraised using the appropriate Critical Appraisal Skills Programme (CASP) tool (CASP Checklists, 2022), the Mixed Methods Appraisal Tool (MMAT) (Hong et al., 2018), and Joanna Briggs Institute (JBI) tool (Critical Apprasial Tools, 2005) depending on the study design. The CASP checklists were used to report on quality of RCT's, cohort, and qualitative studies. Quasi-experimental studies were assessed by the JBI tool and mixed methods, and descriptive studies were assessed using the MMAT. Quality appraisal was used to interpret the findings; therefore, no studies were excluded based on quality appraisal. All quality appraisals were completed independently by two researchers (JG and IT) with 10% checked (CJE, ND, and CH) for consistency.

### Data extraction and synthesis

The data extraction template was informed by the review questions and PRISMA reporting guidance. The template included title, lead author, date of publication, country of study, aim of study, study design, eHealth intervention (type, components, and summary), methods of data collection and analysis, outcomes, implications, and limitations. Data extraction was completed by five researchers (IT, JG, EY, JA, and CH). All extracted data were checked by two researchers (IT and JG).

Quantitative and qualitative data were extracted. Quantitative data on effectiveness were too heterogeneous to pool for meta-analysis. Therefore, we conducted an integrative synthesis to produce a narrative summary (Dixon-Woods et al., 2005) of both quantitative and qualitative data categorized by acceptability or effectiveness. Findings were triangulated in the interpretation (O'Cathain et al., 2010).

## Results

**Figure 1:**
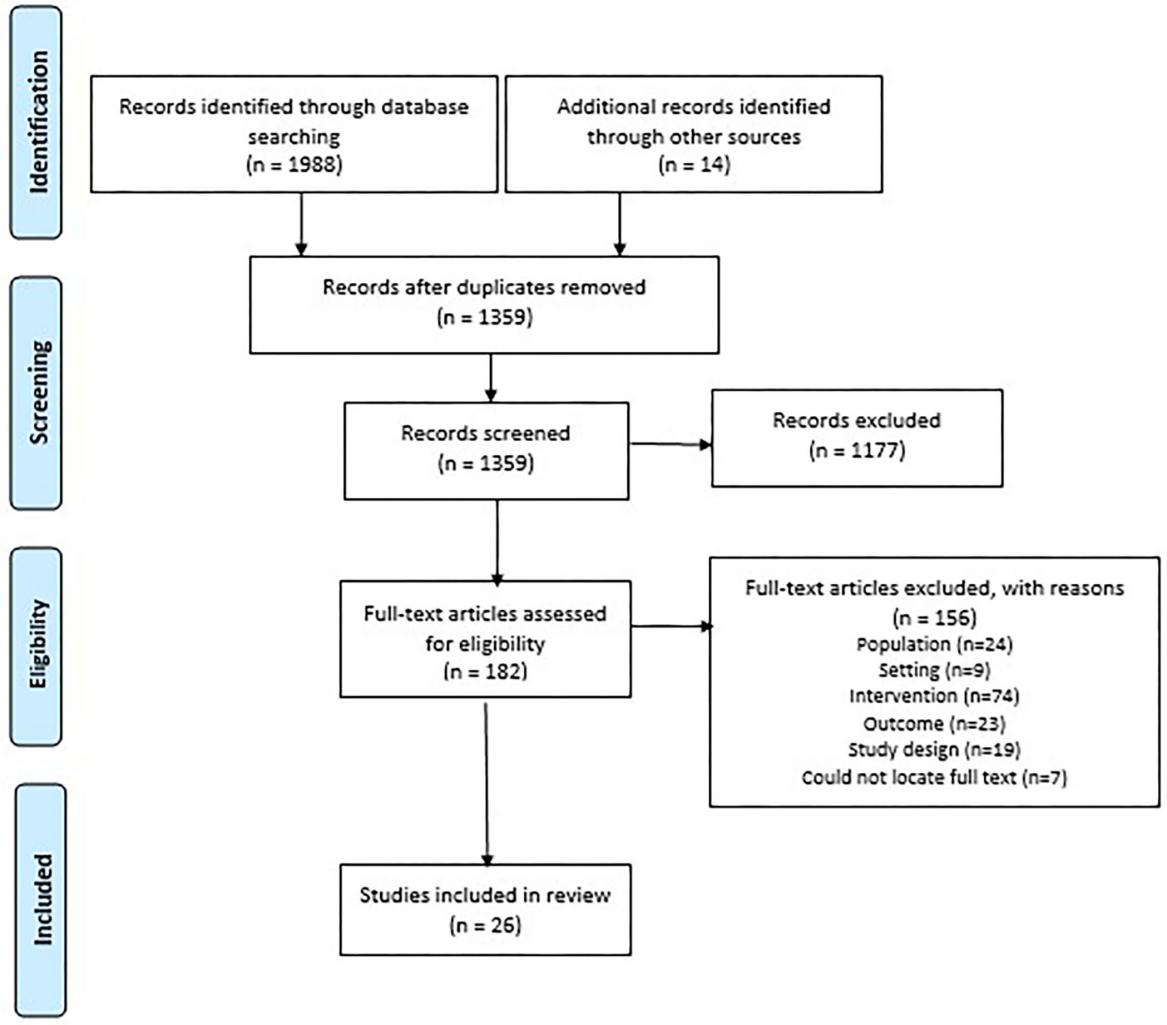
PRISMA flow diagram.

**Figure 2:**
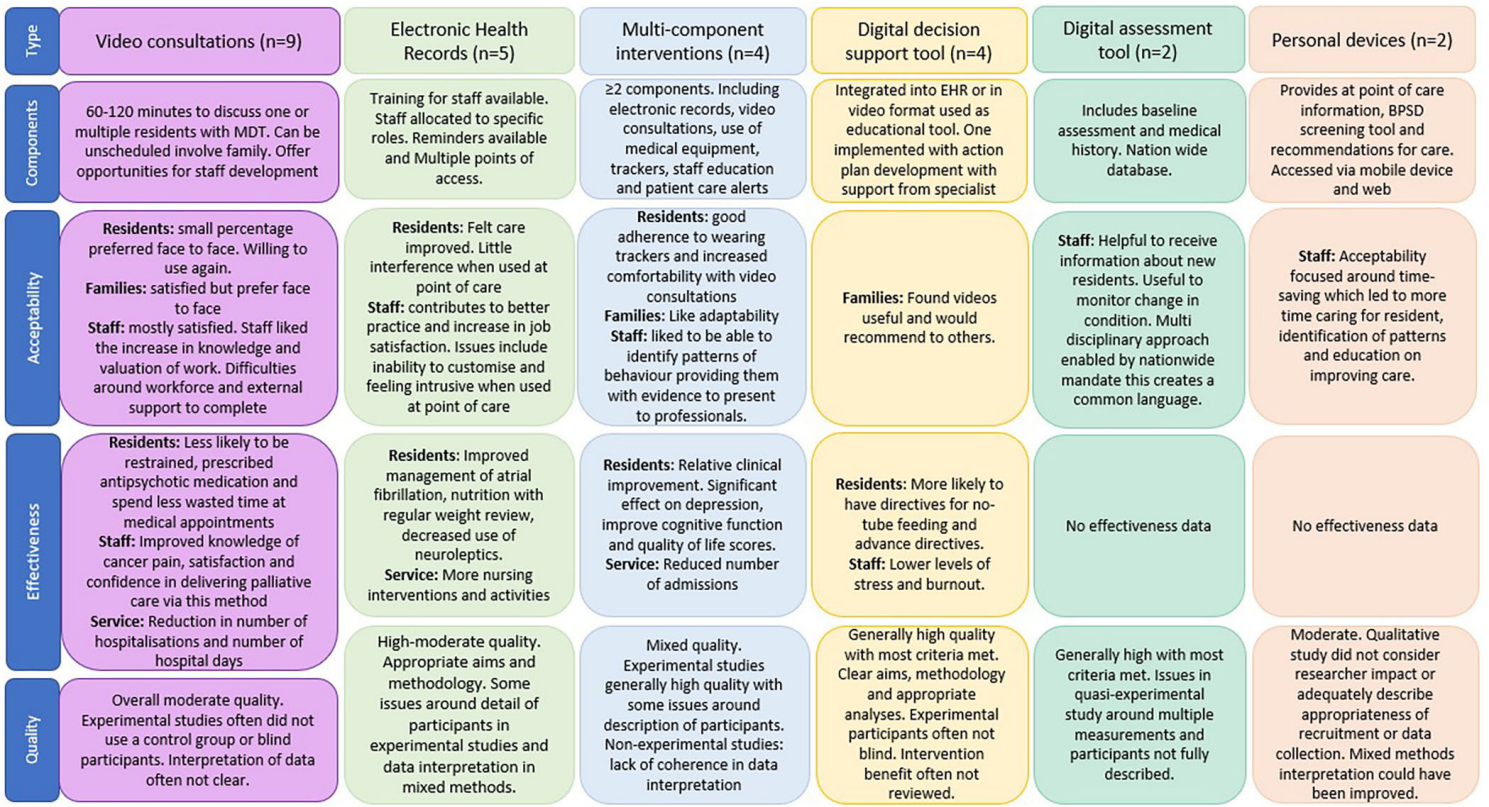
Summary of evidence.

The search strategies yielded 1,988 results. An additional 14 articles were included from alternative sources. Following removal of duplicates, a total of 1,359 articles were screened at title and abstract, and 182 full-text articles were reviewed (Figure 1 PRISMA Flow Diagram). Twenty-six articles reporting twenty-four eHealth interventions were included in this review (summary of evidence in Figure 2).

eHealth interventions to support assessment and decision-making for people with dementia in care homes were categorized as video consultations (*n* = 9) (Lyketsos et al., 2001; Weiner et al., 2003; Wakefield et al., 2004; O'Mahony et al., 2009; Catic et al., 2014; Gordon et al., 2016; Salles et al., 2017; Perri et al., 2020; Piau et al., 2020), electronic health records (EHRs; *n* = 5) (Daly et al., 2002; Krüger et al., 2011; Munyisia et al., 2011; Pillemer et al., 2012; Shiells et al., 2020), multicomponent interventions (constructed of more than one intervention, such as video consultations with digital assessment systems and EHRs; *n* = 4) (Lee et al., 2000; De Luca et al., 2016; De Vito et al., 2020; Wang et al., 2021), digital decision support tools (*n* = 4) (Fossum et al., 2011; Moniz-Cook et al., 2017; Mitchell et al., 2018, 2020), digital assessment tools (*n* = 2) (Vuorinen, 2020; Zahid et al., 2020), and personal devices (*n* = 2) (Qadri et al., 2009; Klein et al., 2018). Studies were categorized as observational exploring the acceptability of the intervention using quantitative (*n* = 2 cross-sectional; *n* = 2 cohort; *n* = 1 descriptive), qualitative (*n* = 5), and mixed methods (*n* = 3), or experimental to evaluate the effectiveness and/or acceptability of interventions (*n* = 5 RCTs; *n* = 8 quasi-experimental).

### Quality appraisal

The included studies were of mixed, but overall high–moderate quality. Full-quality assessment can be found in Table 1. The CASP checklists identified strong reporting of aims, appropriate methodologies, and consideration of ethical issues. The CASP criteria identified weaknesses centered around reporting of benefit, recruitment strategies, and use of blinding. Overall, quasi-experimental studies were of good quality (77.7% met JBI criteria). The cross-sectional study was of moderate quality (50% of JBI criteria met). Mixed methods studies were of moderate quality. The reasoning for mixed methods design was often well-presented within the studies. However, interpretation of results from data was often unclear. Other common issues compromising quality included, confounding factors not considered in the data analysis, comparisons between groups not reported, and insufficient information to assess if quality criteria were adequately met. One descriptive study was assessed as high quality.

**Table 1:** Study characteristics, intervention components, and acceptability of eHealth interventions.

**Author, year, country, study design**	**Population and N**	**Quality assessment (tool)**	**Intervention purpose**	**Intervention components**	**Acceptability**
**Video Consultations**					
Catic et al. (2014) USA, cohort	Residents with dementia (83%; *N* = 47) (severity not provided) at two Veterans Affairs Medical Centers and a state Long-Term Care center. 83% of residents had hypertension, 68% depression, 31% with delirium and 20% with CVA	Clear aims, recruitment, exposure, completeness of follow-up, and application of results. No identification of confounding factors. Cannot tell if outcome was measured and results interpreted accurately (CASP – Cohort Studies).	Remote case-based video-consultation programme called ECHO-AGE to link dementia behavior management experts to nursing home care providers.	**Collaborative working with multidisciplinary team (MDT) (scheduled and unscheduled)** 1.5-h meetings between nursing home staff and team consisting of a geriatrician, geriatrics hospitalist, geriatrics psychiatrist, behavioral neurologist, and community resource specialist to give care management suggestions and ‘take home' recommendations. 2–4 residents discussed per meeting with information provided 48 h prior. MDT available by phone or email for urgent issues. **Family involvement** Families are invited to participate but often do not.	No data.
Gordon et al. (2016) USA, 2:1 matched cohort	Residents with dementia (*N* = 33) (severity not provided) from eleven nursing homes	Clear aims, recruitment, methodology, completeness of follow-up, and interpretation and application of results. (CASP – Cohort Studies).	Remote case-based video-consultation programme called ECHO-AGE to provide access to MDT to reduce use of restraint.	**Collaborative working with multidisciplinary team (MDT) (scheduled)** Biweekly videoconference between teams of frontline nursing home staff and a team of clinical experts, including a geriatrician, geropsychiatrist, behavioral neurologist and social worker. Sessions are 120 min to discuss 3–4 residents.	No data.
Lyketsos et al. (2001) USA, quasi experimental	One Long-Term Care Facility (LCTF) for dementia patients (N not provided) (severity not provided)	22.2% – Clear variables and similar treatment of all participants. Unsure if the participant groups are similar (including at follow-up), measurements were the same and reliable, or if analysis was appropriate. No control group or multiple measurements of outcome. (JBI)	Copper Ridge/Johns Hopkins telemedicine to reduce psychiatric admissions. Residents involved.	**Collaborative working with multidisciplinary team (MDT) (scheduled)** Twice weekly video conferences with MDT. Joint care plans developed and follow-up and continuity of care discussed. **Mobile teleconferencing unit** **Family involvement** Family are invited to participate.	No data.
O'Mahony et al. (2009) USA, quasi- experimental	Two skilled nursing facilities staff (*n* = 133) and residents' with dementia (*n* = 12; score >25 on MMSE) and family members (*n* = 15)	66.6% – Clear methodology with outcomes measured reliably. No control group. Lacking detail on comparison between groups including at follow up. (JBI)	Video consultation to prevent hospital admission through collaborative working with MDT. Resident and family invited to participate.	**Collaborative working with multidisciplinary team (MDT) (scheduled)** 60–90-min video consultations included the institutional palliative care champion at each site, a unit social worker, unit nurses and certified nursing assistants. **Opportunity for care home staff development** Consultations included case-based teaching, discussion, and summarisation. **Summary report/care plan produced by MDT** Within 72 h of the consultation a summary was prepared by the bioethicist, palliative physician, or geriatrician **Family involvement** Scheduled in advance to encourage involvement. Families are given a CD of consultation if cannot attend.	No data.
Perri et al. (2020) Canada, Quasi-experimental	Two long-term care homes. Residents (*n* = 61; mixed population, 73.9% with dementia. Of the 11 expected to take part in video conference, 10 had advanced dementia), family (*n* = 10) and staff (*n* = 22)	88.8% – Clear study variables, comparison of groups, multiple and reliable measurements of outcomes with appropriate analysis. No control group included. (JBI)	Video conferencing to improve access to palliative care specialists. Resident involved.	**Collaborative working with multidisciplinary team (MDT) (scheduled)** Video conference with an interdisciplinary team of palliative care specialists. **Family involvement** Family can attend via video conference.	The majority of family participants (*n* = 9/10) reported overall satisfaction with the videoconference and would be willing to use it again. Family members felt comfortable and respected. High rates of agreement (*n* = 8–10/10) so that the technology, comfort, and privacy were satisfactory. Participants felt videoconferences had improved their experience. However, around 7/10 still indicated that they would have preferred to see the physician in person. Staff satisfaction Likert scale (1 = strongly agree to 5 = strongly disagree) indicated satisfied with videoconferencing (mean 2.1, SD 0.9), willing to have another videoconference if the resident needed (mean 1.8, SD 0.8), and that enhanced provision of palliative care (mean 1.9, SD 0.7). Neutral response in terms of prefer in person care conference (mean 3.1, SD 0.08).
Piau et al. (2020) France, qualitative	Residents (*N* = 90) (dementia diagnosis/severity not reported) who presented with a difficult to manage neuropsychiatric symptom as assessed by family or staff, and staff (n not provided) from ten LCTFs	Clear aims with appropriate methodology, design, data collection, and analysis. Relationship between researcher and participant and ethical issues considered. Not clear if recruitment strategy was appropriate or clear statement of findings. (CASP – qualitative)	Telemedicine consultation to connect staff and residents with specialized units and provide comprehensive and ecological evaluation.	**Collaborative working with multidisciplinary team (MDT) (scheduled)** Consultation with geriatrician from memory expert center trained in neuropsychiatric symptom (NPS) management. Scheduled within 72 h of resident exhibiting NPS. LTCF physicians, nurses, psychologists, and the patient's GP also participated. **Summary report/care plan produced by MDT** A tailored personal plan with a therapeutic strategy was established after the session.	Staff positive perceptions of telemedicine consultation increased by 29 and 36% after implementation. These included involvement of all residents, families, and staff, increased knowledge and better valuation of care home staff work. However, negative perceptions also increased by 7%. This included lack of time and workforce and difficulties in involving the GP and adapting to change. Staff perceptions: *Organizational aspects* Improved access to specialized healthcare but difficulties in involving GP. Also supported access to medical expertise in remote locations but there may be economic issues. *Staff* Staff liked the training, increase in knowledge and upskilling and that their work and skills were valued. Staff also liked the opportunity for collaborative working. However, they did find it difficult to find the time for development, cope with change and feelings of intrusion. *Families* Improved family involvement and built trusting relationships with staff but difficulties around gaining consent. *Residents* Improved evaluation of residents in their own environment, positive impact on NPS and promoted non-pharmacological treatments. Less stress, fewer transfers, and hospitalisations also experienced. No weaknesses reported but introduction of two-tiered medicine is a potential threat.
Salles et al. (2017) France, descriptive study	Residents (*n* = 304) with complex conditions such as dementia (28.4%), chronic wounds (27.8%), and psychiatric disorders (19%), GPs (n not reported) and staff (*n* = 9) from nursing homes (*N* = 39)	Clear research questions, adequate data collection methods, sampling strategy, measurements, and analysis. (MMAT)	Interactive telemedicine to improve access to care and avoid transfer to the emergency room.	**Collaborative working with multidisciplinary team (MDT)** The team comprised: geriatrician, psychiatrist or rehabilitation physician, and a nurse with either behavioral disorders' expertise or wound healing expertise. **Telemedicine MDT tailored to resident** Specialists involved in the telemedicine act were appropriate to residents' condition **Summary report/care plan produced by MDT** Following meeting, a report is sent to GP and NH staff.	Satisfaction measured on Likert scale from 0 (unsatisfied) to 10 (very satisfied). Nursing home teams were overall satisfied with the telemedicine (mean 9.2, SD 0.7), its equipment (mean 7.1, SD 2.3), quality of the report (mean 8.7, SD 1.4) and found propositions easy to follow (mean 8.8, SD 1.5)
Wakefield et al. (2004) USA, cross-sectional survey	Residents (*n* = 62; mixed population, no detail on diagnoses provided), physicians (*n* = 12) and nurses (*n* = 30) from two Veterans Affairs Medical Centers and an LTC center	50% – Exposure and outcomes were measured in a reliable way, and data were analyzed appropriately. However, inclusion criteria, participants, and confounding factors (including how to manage) were not well-described (JBI).	Interactive video consultations to provide timelier access to services that are not available in the facility. Consultations replace traveling over 8 h for hospital consultation. Nurse takes resident to telemedicine room and remains with them.	**Collaborative working with multidisciplinary team (MDT)** Video conferencing between physicians from two Veterans Affairs Medical Centers and staff and residents at a veteran's care home. **Mobile teleconferencing unit** A high-end telemedicine carts (Tele-Doc 5000) with high-resolution monitors, electronic stethoscope, examination light and camera source, remote camera controls and backlit box for transmitting X-rays. A coordinator facilitated consultations.	Overall, 81% (34/68) of residents' evaluations and 99% (64/65) of nursing evaluations indicated satisfaction with consultation process. 88% (63/72) of residents expressed a willingness to use the consultations for future appointments, 69% (50/72) disagreed that they prefer to see the specialist in person, only 14% (10/72) preferred the in-person consultation and 17% (12/72) had no opinion. Physician ratings as good or excellent: Usefulness in developing a diagnosis—78% (59/76) Usefulness in developing a treatment plan—87% (66/76) Quality of the transmission−79% (59/76) Overall satisfaction with equipment, facilities, and format−86% (65/76).
Weiner et al. (2003) USA, RCT	Nursing home (*n* = 1) residents (*n* = 369) (mixed population, no diagnoses provided however *n* = 4 excluded due to cognitive impairment) and physicians (*n* = 6)	Clear aims, randomization, application of results, and stated benefits. Could not clearly account for all participants in conclusion, if the groups were similar or treated equally. No blinding and clinically important outcomes not considered. (CASP – RCT)	Unscheduled video conferencing to increase access to care. Resident involved.	**Collaborative working with multidisciplinary team (MDT) (scheduled and unscheduled)** Physicians from local university with workstations at home to provide some out of hours support **Mobile teleconferencing unit** A wireless 24-h video conferencing workstation with bedside speakerphone and remotely controllable camera. All videos recorded. Workstations also gave remote physicians access to electronic records and previous videos. When physician not available, a non-interactive, scripted batch video could be recorded for later review.	Some participating residents (data not provided) could not comment on the sessions (e.g., due to dementia or sensory impairment), but when asked to rate communication with the doctor, no residents reported that communication was poor or that the communications made healthcare worse than usual. Of 27 videoconferencing sessions, 15 were successful. Physicians were satisfied with 54% (8/15) of videos, 15% (2/15) of videos were rated as neutral and 7.7% as dissatisfied (1/15). Physicians reported no change in workload [mean 4 (1 = workload greater, 7 = workload lighter)] and perceived slight improvement in care [mean 5.5 (1 = worse, 7 = better)].
**Electronic Health Records**					
Daly et al. (2002) USA, Experimental	Registered nurses (*N* = 8) from an LTCF	Clear aims and account of all participants throughout study. Clinically important outcomes were considered. Groups were similar at baseline. Could not tell if the groups were treated equally or randomized. Cannot tell if results applied to local population or if benefit was reviewed. No blinding (CASP–RCT)	Computerized care plans to increase staff productivity, save time, document and improve patient outcomes.	**Equipment provided with training** Computer introduced at site with an 8-h training programme for computer literate staff. **Staff allocated roles** Each nurse allocated 3–5 residents to assess on admission and then every 3 months to 30-month period.	No data.
Krüger et al. (2011) Norway, Before and after cross sectional	Residents (*n* = 513) (mixed population, 76.6% with dementia, severity not reported) and staff (*n* = 272) from seven nursing homes	77.7%–Clear methodology with outcomes measured reliably, follow-up data complete. Control group included. Lacking comparison between groups and multiple measurements of outcome both pre- and postintervention. (JBI)	Electronic patient record system with decision support to support decision-making.	**Reminders** Reminders were placed on patient records, e.g., “patient has diagnosis of atrial fibrillation but is not on warfarin” or “patient has not been weighed in 30 days”.	In the staff user survey, 43% (117/272) reported great or slightly better job satisfaction. 65% (177/272) used application on a daily basis 81% (220/272) exploited reminders when planned the work 90% (245/272) documentation requirements were met 67% (182/272) less time consuming 43% (117/272) increased job satisfaction 72% (196/272) reported that reminders supported them in doing the job 83% (226/272) reported that the application contributed to safer medication.
Munyisia et al. (2011) Australia, mixed methods	Staff at a dementia care special house and a nursing home (pre implementation *n* = 32; post-implementation 6 months, *n* = 25; 18 months, *n* = 25; 31 months, *n* = 15)	Clear questions with appropriate approach. Appropriate participants and measures, data complete and intervention administered as intended. Mixed methods components well-integrated and interpreted. Could not tell if data were interpreted accurately in results, confounders were accounted for, or if adequate rationale for mixed methods design. (MMAT)	Electronic documentation system to improve efficiency, reduce paperwork, improve the quality of nursing data and save caregivers' time.	**Training available** ‘Super users' received weekly training and were responsible for training other staff members. **Implemented in stages** Electronic documentation system included progress notes, care plans, handover sheets, scheduled tasks, and calculation of funding level. Each component introduced in stages.	Staff were mostly happy with the daily progress notes implemented as this allowed for timely updates on resident's care needs. Staff reported progress notes as simple and easy to use. The eHealth intervention was welcomed by the whole team.
Pillemer et al. (2012) USA, non-randomized quasi-experimental	Residents (*N* = 761, intervention group *n* = 428, control group *n* = 333; mixed population, diagnoses not provided) in ten nursing homes	100%–Clear study variables, comparison of groups, multiple and reliable measurements of outcomes with appropriate analysis. Control group included. (JBI)	Health information technology to increase efficiency while offering potential cost savings.	**Point of care access** Electronic health records that were accessible by computer or personal digital assistants so nurses could record and access information from anywhere. The system allowed for scheduling and mobile capture of assessments, interventions, and treatments, and online entry of progress notes by discipline. It further allowed for real-time reporting of sentinel events, quality indicators, and quality measures. **Remote access** The system also included computerized physician order entry, allowing physicians to securely approve orders and access medical records remotely.	Over 70.8% (303/428) of residents agreed the device helped staff to better manage care and they were pleased about it. 69.3% (297/428) felt the device did not interfere with care and over 90% (397/428) reported care had either improved (131/428, 30%) or stayed the same (266/428, 62.2%)
Shiells et al. (2020) Czech Republic, qualitative	Staff (*N* = 21) from three nursing homes	Clear aims, appropriate methodology, design, recruitment, data collection and analysis. Relationship between researcher and participant and ethical issues considered. Clear statement of findings. (CASP – qualitative)	Electronic patient record (EPR) system to assist with documentation processes.	**Staff allocated roles** Different disciplines complete different aspects of the record. **Point of care access** Nurses can access at point of care via a tablet. **Training available** Training available to all staff.	A common issue highlighted by staff was the inability to customize and interoperability. Staff wished to adjust elements of the EPR to meet the needs of the staff and to fit with their practices, including transferability of records from hospital While staff mostly preferred devices accessible at the point of care. Some staff found working at a desktop easier. There were also concerns amongst several staff that the use of technology in the proximity of residents was intrusive and had led to a reduction in the personal aspect of delivering care
**Multicomponent Interventions**					
De Luca et al. (2016) Italy, RCT	Nursing home (*N* = 1) residents (*N* = 59) (mixed population, mean MMSE score of 21.2)	Clear aims, randomized participants, groups treated equally and good application of results including review of benefit. No blinding. Cannot tell if all participants are accounted for throughout, if groups were similar or clinically important outcomes considered. (CASP – RCT)	Telemonitoring with a multimodal approach to improve management of residents.	**Collaborative working** A weekly consultation with neurologist/psychologist via video conferencing. **Medical equipment** Vital signs monitored 3× per week and range of other data recorded, e.g. sound from stethoscope, video and text. **Electronic records** Records accessible to all healthcare professionals involved in care. **Alerts** Alert for when hospitalization required.	No data.
De Vito et al. (2020) USA, mixed methods	Long-term memory care Unit (*N* = 1) residents (*n* = 18) with advanced dementia and staff (n=6)	Clear research questions, appropriate methodology, data collection and interpretation of data. Participants and measures are appropriate. Data are complete and intervention administered as intended. Adequate rationale for mixed methods and components well-integrated. Cannot tell if coherence between qualitative data sources, collection, and interpretation, outputs of integration are interpreted adequately, or confounders considered. Divergences between data not addressed. (MMAT)	Multicomponent Telehealth Care Management Programme to manage and monitor care more effectively and efficiently	**Activity tracker** Residents provided with activity tracker to monitor physical activity and sleep. **Collaborative working** Monthly digital visit with neuropsychologist to assess symptoms *via* iPad. **Care plans** Wellness plans developed and goals set.	Resident daytime average adherence across 6 months – 88% Resident night-time adherence – 58.5–70.9% Staff report that residents attempt to remove tracker when agitated but liked the watch and step counter elements. Staff had positive attitudes toward residents wearing the device. It made providing care easier and more aware of behavior patterns. Upkeep of device generally agreed to be easy and requiring minimal time
Lee et al. (2000) Korea, quasi-experimental	Residents (*n* = 140) (mean MMSE 11.1) and family (*n* = 22) from a nursing home with specialist dementia care facility	88.8% – Clear study variables, comparison of groups, multiple and reliable measurements of outcomes (pre-/post-test) with appropriate analysis. No control group included (JBI)	Telemedicine center inclusive of online database and records, video consultations and medical equipment. The center was developed to expand the capabilities and increase efficiency of healthcare system	**Key staff roles** Each site included a Telemedicine Service Unit that employed one nurse to support clinical interventions. **Education database** The center includes “Silver web” – an online database for information and support for professionals and caregivers. **Electronic records** Online registry database includes follow-up protocol and daily behavior checklist. The database also holds assessments and function tests, caregiver data and admission notes. **Medical equipment** Each site has an X-ray film scanner and scanner for transmission of neuroimaging films. **Remote access** The center also provides flexible and rapid access to remote access to medical expertise regardless of patient or expert location.	Residents responded to the system with slightly tense and frightened facial expressions in the beginning and with more comfortable expressions as they became acquainted with the doctors, within at least several video conferencing sessions. The acceptance by family caregivers and nurses was better due to easy adaptability to the system and no existence of visual or hearing impairments. It took only a few weeks for the nurses at the recipient sites to become accustomed to operating the system. Because they were able to correct the input data up until each record of each patient was completed, the nurses felt relatively comfortable in collecting and inputting the data. They were highly satisfied with video conferencing with the doctor.
Wang et al. (2021) The Netherlands, qualitative evaluation study	Staff (*N* = 7) at a nursing home with specialist dementia care ward	Clear aims, appropriate methodology, design, recruitment, analysis, and statement of findings. Ethical issues considered, but the relationship between researcher and participant not considered. Cannot tell if data collection addressed the research issue. (CASP – Qualitative)	Digital platform with indoor positioning system to personalize BPSD management.	**Activity tracker** Residents and staff wore trackers to monitor location. **Stress monitoring** Staff wrote daily reports and color coded perceived resident stress levels. **Digital platform** Digital platform developed for personalizing BPSD management and creating visualizations to convey a large amount of information quickly	Staff perceived usefulness of the digital platform varied depending on their profession. Caregivers liked that the platform provided evidence for discussion and for confirming feelings. The doctor found insights helpful but required more scientific evidence and the dietician required more information on food and dining. The platform enabled the psychologist to triangulate between data and subjective caregiver reports. The manager of the ward did not find the platform helpful.
**Digital Decision Support Tools**					
Fossum et al. (2011) Norway, quasi-experimental	Residents (*N* = 491) (mixed population, diagnoses not provided) from 46 units (19 specialist dementia units) in 15 nursing homes	100% – Clear study variables, comparison of groups, multiple and reliable measurements of outcomes with appropriate analysis. Control group included. Acknowledged external factors. (JBI)	Computerized Decision Support System to help healthcare professionals to avoid errors and improve clinical practice and efficiency in healthcare.	**Electronic records** The decision support tool was integrated into electronic health records and based on measurements from the Risk Assessment Pressure Scale for PU risk screening and the Mini Nutritional Assessment tool. Evidence-based interventions to support care were suggested from these assessments. **Training available** Tool introduced in 3-day education programme for super users. A further two 45-min educational sessions offered twice, held in nursing homes for all staff. Variety of educational tools were used to motivate staff, such as lectures, discussions, and exercises	No data
Mitchell et al. (2018) USA, cluster RCT	Residents with advanced dementia (*N* = 402; intervention group *n* = 212; control group *n* = 190) from 64 nursing homes	Clear aims, randomization and comparison, and follow-up of participants. No blinding. Cannot tell if groups treated equally, results applied to local population, clinically important outcomes or benefits considered. (CASP – RCT)	ACP video decision support tool to address the shortcomings of traditional ACP discussions.	**Video vignettes to convey options for care** The 12-min video first described the typical features of advanced dementia accompanied by images of an individual with this condition. Three levels of care options were presented: intensive, basic, and comfort care.	Family rated usefulness of videos Very or somewhat helpful 68% (144/212) A little helpful 8.5% (18/212) Unhelpful 23.6% (50/212) Family would recommend the videos to others Definitely/probably 97.1% (205/212) Family who preferred comfort care before watching the video were more likely to find the video unhelpful (40/131, 30.5% vs. 9/80, 11.3%; OR 3.47; 95% CI, 1.58–7.62)
Mitchell et al. (2020) USA, cluster RCT	Residents (*N* = 12,479) (mixed population, 69.4% with advanced dementia) from 360 nursing homes (*n* = 119 intervention, *n* = 241 control)	Clear aims, randomization and comparison, and follow-up of participants. Results applied to local population and clinically important outcomes considered. No blinding. Cannot tell if benefit was considered. (CASP – RCT)	ACP video decision support tool to address the shortcomings of traditional ACP discussions.	**Video vignettes in multiple languages to convey options for care** Five 6–10-min videos in English or Spanish. Topics included: (1) General Goals of Care, (2) Goals of Care for Advanced Dementia, (3) Hospice, (4) Hospitalization, and (5) ACP for Healthy Patients. **Key staff roles** One senior project manager and two ACP video champions per nursing home. Champions were responsible for showing videos to patients and families, (1) within 7 days of admission or readmission, (2) every 6 months, (3) when specific decisions arose (e.g., transition to hospice care), and (4) under special circumstances (e.g., out-of-town family visit) of their choice.	No data.
Moniz-Cook et al. (2017) UK, cluster RCT	Residents with dementia (*n* = 832) (49% with CDR score of 3 indicating severe dementia, 30% with a score of 2) staff (*n* = 609) from 63 care homes	Clear aims, randomization, blinding, and comparison, and follow-up of participants. Results applied to local population and clinically important outcomes considered. Benefit was not reviewed. (CASP–RCT)	Decision support tool with e-learning course for the targeting of individualized or person-centered interventions for challenging behavior in dementia.	**Collates information** The decision support system comprised relevant assessment tools to collect information of key contributory factors associated with challenging behavior **Training available** Three e-learning modules to provide staff with an observational and algorithmic approach to choosing interventions. **Key staff roles** Staff champions also worked with a specialist dementia care therapist, who used a decision support e-tool to develop action plans for a particular behavior that was identified by staff as challenging.	No data.
**Digital Assessment Tools**					
Vuorinen (2020) New Zealand, qualitative interviews	Registered nurses (*N* = 12) from LTCFs (*N* not provided)	Clear aims, appropriate methodology, design, recruitment, data collection and analysis. Relationship between researcher and participant and ethical issues considered. Clear statement of findings. (CASP – qualitative)	International Resident Assessment Instrument for Long-Term Care Facilities (interRAI-LTCF) is a web-based assessment tool designed to comprehensively assess older adults.	Components not described.	Staff reported the most useful aspect of interRAI-LTCF to staff was receiving comprehensive information about the resident's medical history and their baseline nursing assessment. InterRAI-LTCF was also perceived as useful when there was a change in a resident's condition, and the level of care needed to be reviewed by the Needs Assessment team. RNs appreciated a shared interRAI database that is used nationwide which enabled a multidisciplinary approach
Zahid et al. (2020) Canada, case series and quasi-experimental	Nurses and care aides (*N* = 121) working in LTC facilities (*N* = 7)	77.73%–Clear study variables, comparison of groups, reliable measurements of outcomes with appropriate analysis. Control group included. Multiple measurements of outcome not completed. Follow-up not complete and differences between groups not described. (JBI)	Pain Assessment Checklist for Seniors With Limited Ability to Communicate (PACSLAC-II) tool to reduce paperwork and workload	**Literal interpretation of paper version** App version on tablet provided to staff. Instructions provided but app version is a literal interpretation of paper version. **Visualization of results** App produced graph of results over time. **Training available** A web-based training programme of six 10–15-min modules was also implemented with the app.	Staff found the tablet version of the PACSLAC-II user friendly, faster, and easier to access and store the data. Staff did not report any increase in workload and noted any future increase would be worthwhile. Although most staff reported positive experiences, there were some reported issues around connectivity and communication across disciplines, with some staff feeling dismissed by professionals at a higher grade. However, other staff noted that the tool provided a common language across disciplines.
**Personal Devices**					
Klein et al. (2018) Australia, qualitative focus groups and interviews	A regional aged care residential facility. Residents with dementia (*n* = 5) (severity not reported) and nursing staff (n=10)	Clear aims, appropriate methodology, design, and statement of findings. Data analysis not sufficiently rigorous. Ethical issues considered, but the relationship between researcher and participant not considered. Cannot tell if recruitment was appropriate or if data collection addressed the research issue. (CASP – Qualitative)	Nurses' behavioral assistant (NBA), a knowledge-based, interactive eHealth system to assist staff to better respond to behavioral and psychological symptoms of dementia (BPSD).	**Screening tool** A BPSD event screening tool that provided a series of ‘safety' assessment questions around physical health and environmental causes. Held on web dashboard **Recommendations and feedback** Recommendations and feedback about which psychological interventions to employ in response to the specific BPSD events encountered **Web dashboard** A simple web dashboard, graphically displaying the outcomes of the strategies employed **Education** Information was provided on web dashboard **Available on web and mobile app** The prototype NBA system was provided to nursing staff through a secure mobile and web-based application. Mobile phones were also provided to the nursing staff.	Staffs stress levels reduced by ease of use of intervention. NBA allows staff quick access to events and intervention reports. Providing a consistent approach to care. Staff found NBA quicker than current practice allowing them to attend to residents in timelier manner. Staff liked that the NBA gave them the ability to identify patterns and factors associated with BPSD event. Issues included staff forgetting to consult the NBA and confusion around double reporting. Two members of staff also expressed some initial apprehension around using the NBA, recognizing they were less comfortable with mobile technology.
Qadri et al. (2009) USA, mixed methods	Staff (*N* = 25) from three nursing homes	Clear questions with appropriate approach. Appropriate participants, measures and randomization, data complete and intervention administered as intended. Cannot tell if assessors were blind or groups were comparable at baseline. Mixed methods rationale is clear, components well-integrated and interpreted. Outputs of integration not adequately interpreted and divergences in data not addressed. (MMAT)	Personal digital assistant (*via* pocket PC) to implement features of a decision support tool to support staff in managing challenging patient situations.	Components not described.	All participating staff described the tool as “useful” or “helpful”. The tool allowed nurses to focus on the resident's condition and allowed them to learn more about caring for the residents. Nurses were receptive to the use of handhelds containing point-of-patient-care information which was time saving. Nurses found the tools convenient, easy to use, and useful as a reference guide.

ACP, Advanced Care Planning; BPSD, Behavioral and Psychological Symptoms of Dementia; CASP, Critical Appraisal Skills Programme; CDR, Clinical Dementia Rating; CVA, Cerebrovascular accident; EPR, Electronic Patient Record; GP, General Practitioner; interRAI-LTCF, International Resident Assessment Instrument for Long-Term Care Facilities; JBI, Joanna Briggs Institute; LTC(F), long-term care (facility); MDT, Multidisciplinary Team; MMAT, Mixed Methods Appraisal Tool; NBA, Nurses behavioral assistant; NH, nursing home; NPS, Neuropsychiatric Symptoms; OT, Occupational Therapist; PACSLAC-II, Pain Assessment Checklist for Seniors With Limited Ability to Communicate tool; PC, Personal Computer; RCT, Randomized Controlled Trial.

Studies categorized where eHealth intervention is most prominent focus of study.

### Components and acceptability

#### Video consultations

Video consultations were the most common eHealth intervention identified (*n* = 9) (Lyketsos et al., 2001; Weiner et al., 2003; Wakefield et al., 2004; O'Mahony et al., 2009; Catic et al., 2014; Gordon et al., 2016; Salles et al., 2017; Perri et al., 2020; Piau et al., 2020). Video consultations involved an external multidisciplinary team (MDT), care home staff, often residents and, sometimes, their families. Residents, and families, were not involved when consultations were used to discuss more than one resident. The main component of the consultations was to provide care home staff with remote access to MDT expertise and fostered integrated care. MDTs varied in their structure but included professionals such as medical doctors, such as psychiatrist and family physician, nurses, geriatricians, and social workers. The format of consultations varied across studies, for example, length of consultations, scheduling routes, and use of staff champions to initiate and facilitate consultations.

Five studies examined the acceptability of video consultations (Weiner et al., 2003; Wakefield et al., 2004; Salles et al., 2017; Perri et al., 2020; Piau et al., 2020). One study found that overall, families were satisfied with video consultations (86%) with palliative care teams, particularly with the technology, comfort, and privacy, but 70% would still prefer a face-to-face consultation (Perri et al., 2020). However, another study found that only 14% of residents preferred face-to-face hospital appointments, and 88% would be willing to use video consultations again to avoid traveling to the appointment (Wakefield et al., 2004).

Care home staff also demonstrated willingness to use video consultations again, and reported that they enabled timely access to palliative care specialists and enhanced provision of care (Perri et al., 2020), particularly in remote locations (Piau et al., 2020). Importantly, consultations resulted in improved knowledge for care home staff, and staff felt their work was better valued by external professionals (Piau et al., 2020). In addition, staff found that follow-up reports from external professionals were easy to interpret and of good quality (Salles et al., 2017). Care home physicians reported a slight improvement in care and no change in workload (Weiner et al., 2003). However, staff cited challenges of commitment from external professionals, and lack of time and workforce in the care home to participate in consultations (Piau et al., 2020). This hindered integrated working between the care home and external professionals.

#### Electronic health records

Five studies examined the use of EHRs (Daly et al., 2002; Krüger et al., 2011; Munyisia et al., 2011; Pillemer et al., 2012; Shiells et al., 2020), implemented with the intention to improve shared decision-making and increase efficiency of staff time. Common components included training for staff to use EHR (including on equipment), staff allocated specific roles, task reminders, and multiple points of access, such as at the point of care (e.g., resident's beside) and remotely (outside the care home).

Four studies included acceptability data: three for staff (Krüger et al., 2011; Munyisia et al., 2011; Shiells et al., 2020), and one study considering residents (Pillemer et al., 2012). EHRs supported staff to perform their roles better; 72% (*n* = 117) reported that the reminders were useful, 83% (*n* = 226) reported that EHRs contributed to safer use of medication (Krüger et al., 2011), and daily progress notes enabled timely updates on resident's needs (Munyisia et al., 2011). An increase in staff job satisfaction was also observed (43%, *n* = 117) (Krüger et al., 2011). However, frustrations arose around interoperability between services, such as the care home and hospital using different EHR systems. Staff also disliked the inability to customize EHRs to a level that is appropriate for all staff and residents with dementia to avoid input of irrelevant information (Shiells et al., 2020). Preferences on point of access differed among staff, with some preferring at the point of care, with others considering this intrusive and preferring to access the EHR at a desktop computer (Shiells et al., 2020). However, 69% (*n* = 297) of residents felt that staff accessing an EHR in their presence did not interfere with care, and over 70% (*n* = 303) felt that the EHR helped staff to manage care better with 30% (*n* = 131) reporting an improvement in care (Pillemer et al., 2012).

#### Multicomponent interventions

Four studies focused on multicomponent interventions (Lee et al., 2000; De Luca et al., 2016; De Vito et al., 2020; Wang et al., 2021). The included interventions were constructed of two or more components. Components included electronic records, care plans and alerts, staff training (including an education database), video consultations, digital platform, use of medical equipment such as x-ray scanners, and activity trackers. These interventions intended to support integrated working between decision-makers (Lee et al., 2000) and to improve management of a resident's care through monitoring (Lee et al., 2000; De Luca et al., 2016; De Vito et al., 2020; Wang et al., 2021).

Care staff liked the ability to identify patterns of behavior (De Vito et al., 2020) and interventions that provided them with tangible evidence that confirmed their beliefs about a resident's symptoms and concerns to discuss with external professionals (Wang et al., 2021). In one study, a digital platform collated location data alongside qualitative contextual data input by staff to display resident routines over time which were shared with external professionals (Wang et al., 2021).

*For me, since I am not in the ward myself, I normally talk with the caregivers [care home staff]; it is good to see how often he [the resident] is in stress* (from the collated data). [Psychologist] (Wang et al., 2021)

Family and staff both appreciated the adaptability of these interventions, including the ability to amend previously entered data (Lee et al., 2000). No studies examined the acceptability of multicomponent interventions for residents. However, staff reported that residents with dementia showed no discomfort when using activity trackers (De Vito et al., 2020), and became more familiar and comfortable with video consultations with external professionals with repeated use (Lee et al., 2000).

#### Digital decision support tools

Four studies explored decision support tools to enhance communication in advance care planning (Fossum et al., 2011; Moniz-Cook et al., 2017; Mitchell et al., 2018, 2020). Common components included collaborative working with a dementia care therapist and Advance Care Planning (ACP) specialists, ability to populate clinical information (either from integration with EHRs or within itself), training on use readily available and a designated member of staff to initiate and facilitate use.

One study examined the acceptability of digital decision support tools, focusing on family members. Family members watched video vignettes to support advance care planning on care options available to people with advanced dementia. Family members found the videos useful (68%, *n* = 144), and 97% (*n* = 205) of them would recommend the videos to others (Mitchell et al., 2018).

#### Digital assessment tools

Two studies examined digital assessment tools. One study compared a paper and digital app version of the Pain Assessment Checklist for Seniors with Limited Ability to Communicate (PACSLAC-II) tool (Zahid et al., 2020). The app was designed to be a literal interpretation of the paper version with the addition of collating and graphically displaying the results over time. Staff found the app version of the tool to be faster and easier to access and store data. The tool provided care staff with a common language and evidence of change in resident condition to other disciplines, but some staff felt their observations were ignored by external colleagues (Zahid et al., 2020).

*Like I said, we look after these people, we're here more often with these people than we are with our own families. So, we know these people inside and out and so when we say that there's an issue or this person's off or they look like they're having a lot more pain, trust us….the doctor's only here once a week and he spends not very much time with these people and he comes in and he does his two minute assessment and says, ‘they look fine today, no let's hold off.' Really, now we have to go another seven whole days of more documentation for him to say, ‘well, I really don't know, we'll bump them up a little bit.' So, you know what I'm saying, it's the frustration of not being heard. [care staff]* (Feigin et al., 2019)

Vuorinen (2020) evaluated the use of the nationwide mandated, web-based International Resident Assessment Instrument for Long-Term Care Facilities (interRAI-LTCF) to assess older adults' health and care needs. Use of the interRAI-LTCF provided staff with a comprehensive and multidisciplinary history of a resident, with information shared across care facilities, enhancing identification of change in a resident's condition (Vuorinen, 2020).

#### Personal devices

Personal devices were small, computer-like devices that enabled staff to access EHRs or assessments at the point of care. Two studies explored the use of personal devices. One study (Klein et al., 2018) described components as: a screening tool, web dashboard that included an education section, production of recommendations for care and feedback on which interventions to employ, and availability of the tool on multiple formats, such as the web and mobile apps. Both studies found that care home staff were receptive to using personal devices that removed time-wasting paperwork increasing time to address residents' needs (Qadri et al., 2009; Klein et al., 2018). Staff particularly liked the ability to identify patterns and factors associated with distressing symptoms and challenging behavior (Klein et al., 2018) and learning about better ways to care for their residents (Qadri et al., 2009).

### Evidence of effectiveness

Fifteen included studies considered the evidence of effectiveness of eHealth interventions (Table 2). Twelve studies reported resident outcomes (Lee et al., 2000; Daly et al., 2002; O'Mahony et al., 2009; Fossum et al., 2011; Krüger et al., 2011; Pillemer et al., 2012; Catic et al., 2014; De Luca et al., 2016; Gordon et al., 2016; Moniz-Cook et al., 2017; Mitchell et al., 2018, 2020), one study reported on family outcomes (Mitchell et al., 2018), four studies reported staff outcomes (O'Mahony et al., 2009; Moniz-Cook et al., 2017; Perri et al., 2020; Zahid et al., 2020), five studies evaluated outcomes of service delivery (Lyketsos et al., 2001; Daly et al., 2002; Catic et al., 2014; De Luca et al., 2016; Mitchell et al., 2020), and one study reported on economic evaluation (Moniz-Cook et al., 2017).

**Table 2:** Evidence of effectiveness of eHealth interventions by outcome type.

**Author, year, country, design**	**Population and *N***	**eHealth intervention type**	**Aim/Question**	**Control group**	**Outcomes measured (Standardized measure)**	**Effectiveness data**
**Resident outcomes**						
Catic et al. (2014) USA, cohort	Residents with dementia (*N* = 47) at two Veterans Affairs Medical Centers and a state long-term care center	Video consultation	To design, implement, and assess the pilot phase of an innovative, remote case-based video consultation programme called ECHO-AGE that links experts in the management of behavior disorders in patients with dementia to nursing home care providers.	None	Resident improvement Mortality	Where recommendations were followed, 74% of residents clinically improved, compared to 20% where recommendations were not followed (*p* < 0.03). Mortality was significantly lower in residents who improved (4 vs. 50%, *p* < 0.003).
Gordon et al. (2016) USA, 2:1 matched cohort	Residents with dementia (*N* = 33) from eleven nursing homes	Video consultation	To determine the impact of the ECHO-AGE intervention on the quality of care delivered to nursing home residents with dementia across participating facilities. In particular, we aimed to determine whether the intervention reduced physical and chemical restraint use.	Matched controls	Physical restraint (item P0100, E-H in Minimum Data Set (MDS) 3.0) Medications (N0400A in MDS 3.0)	Residents in ECHO-AGE facilities were 75% less likely to be physically restrained (OR = 0.25, *p* = 0.05), 17% less likely to be prescribed antipsychotic medication (although not significant) (OR = 0.83, *p* = 0.07) and 23% less likely to experience a urinary tract infection (OR = 0.77, *p* = 0.01)
O'Mahony et al. (2009) USA, experimental	Two skilled nursing facilities staff (*n* = 133) and residents' with dementia family members (*n* = 15)	Video consultation	(1) To extend hospital-based consultations to local Skilled Nursing Facilities using face-to-face consultations and video consultations; (2) improve quality of life and comfort for residents and families; (3) improve the level of practice and increase staff satisfaction with palliative care content-related knowledge and bioethical analysis.	Compared to face-to-face consultation group	Quality of care (Palliative Outcomes Scale)	Respondents at the video consultation site's rating of time wasted at medical appointments were significantly decreased between baseline and follow-up (*p* = 0.001). Aggregated patient Palliative Outcomes Scale scores were significantly improved at video consultation site (*p* = 0.005)
Daly et al. (2002) USA, Experimental	Registered nurses (*N* = 8) from an LTCF	Electronic health records	To determine how use of standardized nomenclature for nursing diagnosis and intervention statements on the computerized nursing care plan in an LTC facility would affect patient outcomes, and organizational processes and outcomes	Compared to group who completed paper care plans	Function (IADL) Pain (Numerical Rating Scale for Pain) Cognitive ability (MMSE) Medications Weight Pressure ulcers	No significant group differences in patient outcomes – MMSE/level of care/pain score/medications/bowel medications/ weight (statistical data not provided)
Krüger et al. (2011) Norway, before-after study	Residents (*n* = 513) (mixed population, 76.6% with dementia) and staff (*n* = 272) from seven nursing homes	Electronic health records	To study the impact of introducing an electronic patient record system with decision support on the use of warfarin, neuroleptics and weighing of patients in nursing homes and to monitor any negative impact on job satisfaction.	Internal control group	Medications Weight	Warfarin use increased from 3.0% (6/183) to 9.8% (21/205) (*p* = 0.013, 95% CI 1.6–12.1). Neuroleptics decreased from 33% (60/183) to 21.5% (44/205) (*p* = 0.015, 95% CI: 2.3–20.6). Use of other medications did not significantly change. The proportion of patients not weighed for the last 30 days was reduced from 72.6% (133/183) to 16.0% (33/205) (*p* < 0.001, 95% CI: 47.5–64.5).
Pillemer et al. (2012) USA, non-randomized quasi-experimental design	Residents (*N* = 761, intervention group *n* = 428, control group *n* = 333) (mixed population) in 10 nursing homes	Electronic health records	To examine the effects of electronic health information technology (HIT) on nursing home residents	Matched controls	Function Falls Mood Challenging behaviors Mortality	No significant differences found for any variables. No treatment effect for mortality (*p* = 0.09). A negative treatment effect was found in the measure of observed behavior. Residents in treatment facilities experienced an increase in observed disruptive behaviors. There was a reduction in disruptive behaviors over time in the control facilities.
De Luca et al. (2016) Italy, RCT	Nursing home (*N* = 1) residents (*N* = 59)(mixed population, mean MMSE score of 21.2)	Multicomponent interventions	The purpose of this study was to develop a novel Sicilian Tele-Health-Care model and to evaluate its effectiveness	Standard care control group	Mood (GDS and BPRS) Function (ADL and IADL) Vital signs Quality of life (EuroQoL VAS) Alzheimer's Severity (BANSS)	Experimental group demonstrated significant reductions in depression (*p* < 0.01) and mood (BPRS) (*p* < 0.05) scores and more significant improvement in quality of life (*p* < 0.001) compared to the control group. Blood pressure (*p* < 0.001) and heart rate (*p* < 0.05) were also significantly reduced.
Lee et al. (2000) Korea, quasi experimental	Residents (*n* = 140) and family (*n* = 22) from a nursing home with specialist dementia care facility	Multicomponent interventions	To examine the acceptance, reliability, and clinical outcome of our telemedicine service	None	Challenging behaviors (Daily behavior checklist) Sleep disturbances (daily behavior checklist)	46% (64/140) of nursing home residents showed relative clinical improvement
Fossum et al. (2011) Norway, quasi-experimental design	Residents (*N* = 491) (mixed population) from 46 units (19 specialist dementia units) in 15 nursing homes	Digital decision support tool	To evaluate the effects on the risk for and prevalence of pressure ulcers (PUs) and malnutrition when implementing a Computerized Decision Support System	Standard care control	Pressure ulcers (RAPS) Nutritional status (MNA)	No statistically significant effects between the two intervention groups and one control group when comparing the prevalence of PUs before and after the intervention (*p* = 0.31), the prevalence of residents with adequate nutritional status (MNA ≥ 24) and those with malnutrition (MNA < 17) between groups and occasions (2007 and 2009) (*p* = 0.19).
Mitchell et al. (2018) USA, cluster RCT	Residents with advanced dementia (*N* = 402; intervention group *n* = 212; control group *n* = 190) from 64 nursing homes	Digital decision support tool	To test whether an ACP video (vs. usual care) impacted documented advance directives, level of care preferences, goals-of-care discussions, and burdensome treatments	Control group participated in usual ACP practices	Do Not Hospitalize (DNH) directives Forgo tube feeding and intravenous hydration directives Documented goals of care discussions Burdensome treatments	Overall, residents in the intervention arm were more likely to have documented advance directives for no tube-feeding at 6 months (AOR, 1.79; 95% CI, 1.13–2.82) and at all other time points, and documented goals-of-care discussions at 3 months (AOR, 2.58; 95% CI, 1.20–5.54). No differences in proportion of residents with DNH directives between arms at 6 months (AOR, 1.08; 95% CI, 0.69–1.69). Where proxies preferred comfort care, residents in the intervention group were significantly more likely to have directives for do not hospitalize and no-tube feeding (AOR, 2.68; 95% CI, 2.68–5.85), and no tube-feeding alone (AOR, 3.39; 95% CI, 1.62–7.11). No differences in advance directives to withhold intravenous hydration and number of burdensome treatments between arms.
Mitchell et al. (2020) USA, cluster RCT	Residents (*N* = 12,479) (mixed population, 69.4% with advanced dementia) from 360 nursing homes (*n* = 119 intervention, *n* = 241 control)	Digital decision support tool	ACP video decision support tool to address the shortcomings of traditional ACP discussions.	Control group participated in usual ACP practices	Burdensome treatments	The proportion experiencing burdensome treatments did not significantly differ between groups
Moniz-Cook et al. (2017) UK, cluster RCT	Residents with dementia (*n* = 832) staff (*n* = 609) from 63 care homes	Decision support tool and staff development	To evaluate the clinical effectiveness and cost-effectiveness relative to usual care of an online application to enable care home staff to understand the function of challenging behavior in people with dementia and support them accordingly	Usual care controls	Challenging behaviors (NPI and CMAI) Emotion (NPI and CBS) Quality of life (EQ-5D and QoL-AD)	No differences between treatments groups on the primary outcome measure, the Neuropsychiatric Inventory, frequency and severity scores. No other outcome measure showed significant differences.
**Family outcomes**						
Mitchell et al. (2018) USA, cluster RCT	Residents with advanced dementia (*N* = 402; intervention group *n* = 212; control group *n* = 190) from 64 nursing homes	Digital decision support tool	To test whether an Advance Care Planning (ACP) video (vs. usual care) impacted documented advance directives, level of care preferences, goals-of-care discussions, and burdensome treatments	Control group participated in usual ACP practices	Preference for treatment	ACP videos had demonstrated no change in proportion of proxies preferring comfort care. No differences in proportion of proxies who preferred comfort care between intervention and control groups
**Staff outcomes**						
O'Mahony et al. (2009) USA, Two group quasi experimental project pilot, pre- post	Two skilled nursing facilities staff (*n* = 133) and residents' with dementia family members (*n* = 15)	Video consultation	(1) To extend hospital-based consultations to local Skilled Nursing Facilities using face-to-face consultations and video consultations; (2) improve quality of life and comfort for residents and families; and (3) improve the level of practice and increase staff satisfaction with palliative care content-related knowledge and bioethical analysis.	Compared to face-to-face consultation group	Knowledge	Staff knowledge improved particularly in management of cancer pain (*p* = 0.03). Aggregated staff and patient Palliative Outcomes Scale scores were significantly improved at video consultation site (*p* = 0.005)
Perri et al. (2020) Canada, mixed-methods evaluation of pilot implementation	Two long-term care homes. Residents (*n* = 61) (mixed population, 73.9% with dementia), family (*n* = 10) and staff (*n* = 22)	Video consultation	Evaluating whether integration of early palliative care specialist consultation into an LTCH would be feasible through the implementation of videoconferencing during routine interdisciplinary care conferences.	None	Confidence	Staff confidence in delivering palliative care through telemedicine significantly increased (*p* = 0.0021)
Zahid et al. (2020) Canada, case series and quasi-experimental	Nurses and care aides (*N* = 121) working in LTC facilities (*N* = 7).	Digital assessment tool	To compare a newly developed tablet app version of the PACSLAC-II with the original paper-and-pencil version	Compared to group who completed paper-and-pencil version	Stress (including workload) (NSS) Burnout (including emotional exhaustion and depersonalization) (MBI)	The tablet app version was associated with lower levels of stress and burnout in staff. Staff in paper-and-pencil groups (only or after tablet app) experienced significantly higher levels of emotional exhaustion and workload compared to those in tablet app groups (only or after pencil-and-paper). Staff in pencil-and-paper only condition reported significantly higher levels of depersonalization.
Moniz-Cook et al. (2017) UK, cluster RCT	Residents with dementia (*n* = 832) staff (*n* = 609) from 63 care homes	Decision support tool and staff development	To evaluate the clinical effectiveness and cost-effectiveness relative to usual care of an online application to enable care home staff to understand the function of challenging behavior in people with dementia and support them accordingly	Usual care controls	Emotions (including burnout) (MBI and EQ-5D) Attitude (ADQ) Self-efficacy (SES)	No significant effects of the intervention were found in the staff measures for either of the models.
**Service delivery outcomes**						
Catic et al. (2014) USA, cohort	Residents with dementia (*N* = 47) at two Veterans Affairs Medical Centers and a state long-term care center	Video consultation	To design, implement, and assess the pilot phase of an innovative, remote case-based video consultation programme called ECHO-AGE that links experts in the management of behavior disorders in patients with dementia to nursing home care providers.	None	Hospitalisations	Hospitalisations were less common in patients where recommendations had been followed (29 vs. 60%)
Lyketsos et al. (2001) USA, quasi experimental	One long-term care facility (LCTF) for dementia patients (N not provided)	Video consultations	To test the impact of Copper Ridge/Johns Hopkins telemedicine project on reduction of psychiatric admissions	None	Hospitalisations	Video consultations reduce the number of hospitalisations and number of hospital days, but did not reduce the mean length of stay. No test of significance.
Daly et al. (2002) USA, Experimental	Registered nurses (*N* = 8) from an LTCF	Electronic health records	To determine how use of standardized nomenclature for nursing diagnosis and intervention statements on the computerized nursing care plan in an LTC facility would affect patient outcomes, and organizational processes and outcomes	Compared to group who completed paper care plans	Level of care Nursing interventions and activities	Significantly more nursing interventions (*P* = 0.001) and activities (*p* = 0.007) in the computerized care plan group. Computerized care plans took longer to develop at each time point.
De Luca et al. (2016) Italy, RCT	Nursing home (*N* = 1) residents (*N* = 59) (mixed population, mean MMSE score of 21.2)	Multicomponent intervention	The purpose of this study was to develop a novel Sicilian Tele-Health-Care model and to evaluate its effectiveness	Standard care control group	Admission to healthcare service	Admission to healthcare service was higher (X^2^ = 3.96, *p* < 0.05) in the control group (8/27) than in the experimental group (3/32).
Mitchell et al. (2020) USA, cluster RCT	Residents (*N* = 12,479) (mixed population, 69.4% with advanced dementia) from 360 nursing homes (*n* = 119 intervention, *n* = 241 control)	Digital decision support tool	ACP video decision support tool to address the shortcomings of traditional ACP discussions.	Control group participated in usual ACP practices	Hospital transfers Hospice enrolment	There was no significant reduction of hospital transfers per 1,000 person-day alive between the intervention (3.7; SE, 0.2; 95% CI, 3.4–4.0) and control group (3.9; SE, 0.3; 95% CI, 3.6–4.1) (rate difference, −0.2; SE, 0.3; 95% CI, −0.5 to 0.2). No significant difference found for hospital transfers or hospice enrolment between those with advanced illness and those without.
**Economic outcomes**						
Moniz-Cook et al. (2017) UK, cluster RCT	Residents with dementia (*n* = 832) staff (*n* = 609) from 63 care homes	Decision support tool and staff development	To evaluate the clinical effectiveness and cost-effectiveness relative to usual care of an online application to enable care home staff to understand the function of challenging behavior in people with dementia and support them accordingly	Usual care controls	Health and social care use (CSRI) Cost-effectiveness and utility (EQ-5D and MBI)	No significant differences in costs or staff reported quality-adjusted life-years between groups. Mean cost was £331 less in the intervention group.

ACP, Advance Care Planning; ADL, Activities of Daily Living; ADQ, Approaches to Dementia Questionnaire; AOR, Adjusted Odds Ratio; BANSS, Bedford Alzheimer Nursing Severity Scale; BPRS, Brief Psychiatric Rating Scale; CBS, Challenging Behaviors Scale; CI, Confidence Interval; CMAI, Cohen–Mansfield Agitation Inventory; CSRI, Client Services Receipt Inventory; DNH, Do Not Hospitalize; GDS, Geriatric Depression Scale; EuroQoL-Vas, EuroQoL Visual Analog Scale; IADL, Instrumental Activities of Daily Living; MBI, Maslach Burnout Inventory; MDS, Minimum Data Set; MMSE, Mini Mental State Exam; MNA, Mini Nutritional Assessment; NPI, Neuropsychiatric Inventory; NSS, Nursing Stress Scale; PU, Pressure ulcer; RAPS, Risk Assessment for Pressure Sores; SES, Self-Efficacy Scale.

#### Resident outcomes

The eHealth interventions were shown to improve monitoring of resident outcomes which led to changes in prescribing. Studies of eHealth interventions using EHRs and video consultations demonstrated improved outcomes for residents, with those in the intervention groups less likely to be prescribed antipsychotic medications (33 vs. 21.5%, *p* = 0.015, 95% CI: 2.3–20.6) compared with internal controls (Krüger et al., 2011). EHRs with clinical care reminders led to increased use of warfarin (*p* = 0.013, 95% CI 1.6–12.1) and monitoring of residents' weight (*p* < 0.001, 95% CI: 47.5–64.5) (Krüger et al., 2011).

Video consultations also improved resident outcomes over time, although some specific improvements were not detailed (Lee et al., 2000; O'Mahony et al., 2009; Catic et al., 2014). Educational video consultations with an MDT led to a reduction in physical restraint (odds ratio, OR = 0.25, *p* = 0.05) and in urinary tract infections (OR = 0.77, *p* = 0.01) compared to matched controls (Gordon et al., 2016). Residents were also less likely to report time wasted at appointments when assessed by professionals in video consultations (*p* = 0.001) compared to those participating in face-to-face consultations (O'Mahony et al., 2009). One multicomponent intervention comprising 59 participants, that included video consultations, demonstrated significant reductions in depression (*p* < 0.01), mood (*p* < 0.05), blood pressure (*p* < 0.001), and heart rate (*p* < 0.05) and increase in quality of life (*p* < 0.001) compared to standard care controls (De Luca et al., 2016).

Mitchell et al. (2018) found that introducing an ACP decision support tool could support ACP for people with advanced dementia (*N* = 402). Residents whose family members watched an ACP video were more likely to have advance directives for no-tube feeding and documented goals-of-care discussions than residents whose family members who participated in usual ACP practices. However, the intervention did not result in a change in the overall proportion of Do Not Hospitalize directives or burdensome treatments (Mitchell et al., 2018, 2020). Do Not Hospitalize directives were only increased in the intervention group when family members preferred comfort care and when combined with no-tube feeding directives (72.2 vs. 52.8%, a OR, 2.68; 95% CI, 2.68–5.85) (Mitchell et al., 2018).

#### Family outcomes

One study found that ACP decision support tools did not change the proportion of family members preferring comfort care compared to those who participated in usual ACP practices (Mitchell et al., 2018).

#### Staff outcomes

The eHealth interventions were shown to improve care home staff knowledge, confidence and wellbeing. Video consultations with MDTs led to improved knowledge (*p* = 0.03) (O'Mahony et al., 2009) and confidence to deliver palliative care in this way (*p* = 0.002) (Perri et al., 2020). In addition, a reduction in paperwork due to digitized assessment tools resulted in lower levels of stress and burnout for staff (Zahid et al., 2020).

#### Service delivery outcomes

Three interventions that included video consultations with MDTs showed evidence of effectiveness at reducing the number of admissions to hospital in intervention groups (X^2^ = 3.96, *p* < 0.05) (Lyketsos et al., 2001; Catic et al., 2014; De Luca et al., 2016), whereas decision support tools did not have any effect on hospital transfers or hospice enrolment (Mitchell et al., 2020).

An electronic health record that included a computerized care plan to support nurses to regularly monitor residents lead to significantly more nursing interventions (*p* = 0.001) and activities (*p* = 0.007) (Daly et al., 2002).

#### Economic outcomes

A decision support tool with staff development intervention was shown to cost £331 less than usual care. However, this was not a significant difference (Moniz-Cook et al., 2017). No other studies included economic evaluations.

## Discussion

Twenty-six studies were identified evaluating the acceptability and/or effectiveness of eHealth interventions to support assessment and decision-making for people living with dementia in care homes. Seventeen studies reported acceptability data, and fifteen reported effectiveness data. The quality of studies was mixed but mostly moderate to high. There was heterogeneity across all aspects of included studies, from the interventions to outcomes evaluated. The studies also varied in their purpose of using the eHealth intervention, from increasing staff productivity to managing symptoms and improving care, including palliative care. Although some studies showed evidence of effectiveness, most studies had mixed or no effect on the stated outcomes. Only one study considered economic evaluation with focus on cost of an eHealth intervention compared with usual care and showed inconclusive findings. No studies considered cost-effectiveness of the eHealth interventions.

Findings from this review indicate that eHealth interventions that include a video consultation component were most likely to be acceptable to staff and residents. Video consultations suggested effectiveness in outcomes such as reducing use of physical restraint by 75% (Gordon et al., 2016) and hospitalisations (Lyketsos et al., 2001; Catic et al., 2014), which may reduce stress and increase comfort by enabling the person with dementia to remain in their usual place of care. A recent study found that hospitalisations increase steeply in the last year of life for people with dementia (Yorganci et al., 2022); hence, it is vital to utilize interventions to reduce or avoid hospitalisations. Residents with dementia were often able to participate in video consultations and showed satisfaction in the method of consultation and reduction in time wasted at appointments (O'Mahony et al., 2009). Resident outcomes of optimal prescribing of medications improved through use of video consultations compared with matched controls (Gordon et al., 2016). Residents' families were often invited to participate in video consultations. This increased feelings of being respected and trusting relationships (Perri et al., 2020). Video consultations significantly improved staff outcomes around knowledge and confidence. These findings corroborate findings from our related review on implementation of eHealth in care homes (Gillam et al., 2022). This identified that successful implementation requires staff training to increase knowledge, in turn improving staff and resident outcomes.

It is likely that video consultations were most acceptable to staff and residents as they facilitated integrated working with external professionals. Similar findings are reported from research on case-conferencing for people with dementia (Phillips et al., 2013). For staff, video consultations provided a dedicated space for ongoing, practical support and training with external professionals to manage residents' often multiple and complex care needs (Davies et al., 2011; Rivett et al., 2019). This ongoing supportive integration with external professionals provided opportunities for development akin to training which, when sustained, can build staff expertise and confidence (Rivett et al., 2019; Dowling et al., 2020). A workforce that is well-educated and supported provides better quality of care, including toward the end of life (Froggatt, 2000). Furthermore, eHealth interventions were acceptable to staff when they provided them with a common language and evidence of their intrinsic knowledge about a resident's condition to communicate with external professionals. For example, multicomponent interventions were preferred by care home staff when they produced a good visual representation or report of residents' condition overtime and shared with external professionals in video consultations. When visual representations of data are well-produced and interpreted, they contribute to the intervention's success by communicating data to all parties effectively and succinctly. This common language improved confidence, enabling staff to feel empowered and that their care was valued by external professionals. Empowerment was strengthened through video consultations that provide the opportunity for clarification of roles and shared decision-making with key professionals (Phillips et al., 2013). Feeling dismissed by and lack of commitment from external professionals was cited as a challenge to using an eHealth intervention in this review. This failure to recognize care home staff expertise is a known barrier to integrated working (Davies et al., 2011).

This review found evidence of positive outcomes from eHealth interventions that were supported by a structural level of integration between care homes and external professionals. Empowering care home staff is enhanced through investment in infrastructure, specifically around adequate resource and enabling positive leadership (Laschinger et al., 2013) as an individual's desire to participate in integrated working is often insufficient alone to improve outcomes (Goodman et al., 2016). For example, a nationwide mandate to complete eHealth intervention provided staff with a comprehensive, multidisciplinary history of the resident, enabling better care (Vuorinen, 2020). The Enhanced Care in Care Homes framework in England advocates for the use of eHealth interventions and integrated, multidisciplinary care, particularly with a mental health specialist, to support management of care for people with dementia (NHS England, 2016). In addition, the European Association of Palliative Care advocate for a multidisciplinary approach and utility of eHealth interventions as aspects of core competencies required by nursing homes (Gamondi et al., 2013a,b). These initiatives may work toward improving equity of provision of eHealth interventions by ensuring core components around integrated working are embedded in care homes at a structural level, such as access to specialists. With the increase in the use of eHealth interventions in care homes due to the COVID-19 pandemic, it is important that access is equitable for all (Warmoth et al., 2022).

This review found little evidence concerning resident and family acceptability of eHealth interventions to support assessment and decision-making and effectiveness on family outcomes. Although many of the eHealth interventions included the resident in their activity, this review found five studies that considered acceptability for residents', with only three studies that consulted with residents directly, and only one that considered the views of family members. Two studies in this review found that residents with dementia appreciated video consultations as they reduced time spent traveling to appointments (Wakefield et al., 2004; O'Mahony et al., 2009). People with dementia value participating in decision-making about their care (Daly et al., 2018) and play an important role in the development of eHealth interventions to support their care (Span et al., 2013). Where ability to participate is limited or compromised, researchers should seek solutions to enable people with dementia to participate, this might include seeking a personal proxy. Solutions have been offered in the MORECare_Capacity Statement (Evans et al., 2020). It is particularly important that residents with dementia participate in the development of eHealth interventions as the unprecedented uptake in use of eHealth interventions during caused by the COVID-19 pandemic and after is likely to remain (Shepherd et al., 2019).

### Limitations

The review has demonstrated the acceptability and potential of eHealth interventions to enhance assessment and decision-making for residents with dementia in care homes and improve outcomes. However, the review has limitations. We adopted a broad inclusion criteria of effectiveness data, thereby including uncontrolled studies due to the limited number of controlled trials in this emergent field of eHealth. We recognize that the inclusion of uncontrolled studies may have introduced some biases in the findings. In addition, the review was limited by the heterogeneity of the studies included meaning we were unable to perform any meta-analyses to draw strong conclusions and limited this review to an integrative synthesis and narrative summary of the evidence. We wish to acknowledge that all, except one, studies were conducted in the Americas or Europe, and all were conducted in high-income countries. This leads to a gap in knowledge about acceptability and effectiveness of eHealth interventions for people with dementia in care homes in other cultures. We propose that future research explores the acceptability and effectiveness of eHealth in low- and middle-income countries and non-Western cultures. Finally, gray literature was not included in this review leaving potential for publication bias. Gray literature was reviewed and excluded due to limited relevant data available. This may have led to exclusion of some relevant data.

## Conclusions

Findings from this review suggest that eHealth interventions are overall acceptable for staff and have potential to improve outcomes. Most evidence was found for video consultations. Interventions with a video consultation component were shown to be effective at improving resident and staff outcomes. Video consultations with external MDTs were particularly well-received by staff to strengthen knowledge and confidence through regular, supportive, and practical training opportunities. EHRs, digital assessment tools, and personal devices support consistent assessment and monitoring of symptoms over time to identify patterns and improve care and outcomes. Multicomponent interventions build on the work of EHRs by providing enhanced data collection methods, contributing to a detailed assessment, and monitoring. The digitisation of assessment and decision-making tools provides an efficient way of working with a common language for care home staff to communicate with external professionals. Commitment from care home staff can support implementation, but structural level commitment, through supportive infrastructure, and commitment from external professionals is also required to ensure equity of provision to eHealth interventions and access to external professionals. It is important that future research explores the acceptability of eHealth interventions for residents with dementia and their families, how eHealth might affect family outcomes, and if eHealth is a cost-effective way of improving outcomes for residents with dementia. Further work should also focus on eHealth interventions for residents with dementia in low- and middle-income countries.

## Data availability statement

The original contributions presented in the study are included in the article/Supplementary material, further inquiries can be directed to the corresponding author.

## Author contributions

IT, JG, ND, CE-S, and CJE designed the study. IT, JG, and CH contributed to the data screening, extraction, and analysis. VV reviewed the evidence on effectiveness. IT wrote the manuscript. All authors contributed to the revisions and approved the final manuscript.

## Funding

The programme EMBED-Care – Empowering better end-of-life dementia care was funded by the Economic and Social Research Council (ESRC)/National Institute for Health and Care Research (NIHR), Dementia Initiative 2018 (Grant reference number ES/S010327/1), EMBED-Care is a joint study between University College London (UCL) and King's College London (KCL). IT was funded by NIHR Pre-Doctoral Fellowship (NIHR301985). CJE was funded by a Health Education England/NIHR Senior Clinical Lectureship (ICA-SCL-2015-01-001). ND was supported by a fellowship from the Alzheimer's Society, United Kingdom (Grant Number AS-JF-16b-012). The EMBED-Care programme was supported by the NIHR Applied Research Collaborations for South London, and Kent, Surrey, and Sussex.

## Conflict of interest

The authors declare that the research was conducted in the absence of any commercial or financial relationships that could be construed as a potential conflict of interest.

## Publisher's note

All claims expressed in this article are solely those of the authors and do not necessarily represent those of their affiliated organizations, or those of the publisher, the editors and the reviewers. Any product that may be evaluated in this article, or claim that may be made by its manufacturer, is not guaranteed or endorsed by the publisher.

## Author disclaimer

The views expressed were those of the authors and not necessarily those of the ESRC, NIHR, or the Department of Health and Social Care.
