# Supplementary material for: The acceptability and effectiveness of eHealth interventions to support assessment and decision-making for people with dementia living in care homes: A systematic review
Source: Front Dement. 2022 Sep 13;1:977561. doi: 10.3389/frdem.2022.977561 (PMC11285551; doi:10.3389/frdem.2022.977561)
Supplement: Supplementary file 2 [file Data_Sheet_2.pdf]

Supplementary material 2. Search Strategy

Database	Concept	MeSH terms	Key words
**CINHAL**	Dementia	(MM "Dementia+") OR (MH "Nursing Home Patients") OR (MH "Dementia Patients")	“dementia*” OR “senile dementia” OR “vascular dementia” OR “Alzheimer* or PWD
	Care home	**(**MH "Nursing Homes) OR (MH "Residential Care")	"nursing homes” or “nursing home” or “care home” or “care homes” or “long term care” or “residential care” or “aged care facility"
	eHealth	(MH "Telecommunications+") OR (MH "Telehealth+") OR ("Electronic Health Records") or (MH "Health Information Systems+")	“e#tool” or “e#health” or “digital*” or “health informat*” or “technology*” or “telehealth*” or “telecon*” or “telemed*” or “telediag*” or “telemonitor*” or “telecom*” or “telecare” or “information communication tech*” or “interactive health communication” or “electronic shared record” or “electronic communic*” or "electronic health” or “online*” or “internet” or “video decision support” or “videocon*” or “video-con*” or “mobile* or “m?hteleealth” or “app” or “web*” or “virtual”
	Intervention	(MH "Geriatric Assessment") OR (MH "Clinical Assessment Tools") OR (MH "Decision Making, Clinical") OR (MH "Nursing Process+") OR (MH "Quality of Care Research")	“assessment” or “decision*” or “communicat*” or “intervention” or “delivery of care” or “support” or “advis* or “program” or “educat* or “train*” or “coordinat*” or “integ*”

**MEDLINE**	Dementia	exp Dementia/ or Cognitive Dysfunction/	("dementia*" or "senile dementia" or "vascular dementia" or "alzheimer*" or "PWD")
	Care home	exp Nursing Homes/ or Homes for the Aged/ or Long-Term Care/	("nursing homes" or "nursing home" or "care home" or "care homes" or "long term care" or "residential care" or "aged care facility")
	Telehealth	exp Telemedicine/ or exp Telecommunications/ or Mobile Applications/ or exp Medical Records Systems, Computerized/	(e?tool or e?health or digital or health informat* or technolog* or telehealth* or telecon* or telemed* or telediag* or telemonitor* or telecom* or telecare or information communication tech* or interactive health communication or electronic shared record or electronic communic* or electronic health or online or internet* or video decision support or Videocon* or video-con* OR mobile* or m?health or app or web* or virtual)
	Intervention	Geriatric assessment/ or Nursing assessment/ or exp Decision Making, Computer-Assisted/ or Internet-Based Intervention/ or "continuity of patient care"/ or Clinical Decision-Making/	assessment or decision* or communicat* or intervention or care delivery or support or advis* or program or educat* or train* or coordinat* or integ*
**PsycINFO**	Dementia	exp dementia/	dementia* OR senile dementia OR vascular dementia OR Alzheimer* or PWD
	Care home	Long Term Care/ or exp nursing homes/ or	nursing homes or nursing home or care home or care homes or

		residential care institutions/	long term care or residential care or aged care facility
	eHealth	exp telecommunications media/ or exp telemedicine/ or exp electronic health records/ or exp electronic health services/ or exp videoconferencing/ or exp health information technology/ or exp video based interventions/	e?tool or e?health or digital or health informat* or technolog* or telehealth* or telecon* or telemed* or telediag* or telemonitor* or telecom* or telecare or information communication tech* or interactive health communication or electronic shared record or electronic communic* or electronic health or online or internet* or video decision support or Videocon* or video-con* OR mobile* or m?health or app or web* or virtual
	Intervention	exp geriatric assessment/ or Neuropsychological Assessment/ or exp "Clinical Judgment (Not Diagnosis)"/ or exp intervention/ or exp "continuum of care"/	assessment or decision* or communicat* or intervention or care delivery or support or advis* or program or educat* or train* or coordinat* or integ*
**Embase**	Dementia	exp dementia/	dementia* OR senile dementia OR vascular dementia OR Alzheimer* or PWD
	Care home	exp nursing home/ or exp home for the aged/ or exp residential home/	nursing homes or nursing home or care home or care homes or long term care or residential care or aged care facility
	eHealth	exp telemedicine/ or exp telehealth/ or exp electronic health record/ or web-based intervention/ or exp videoconferencing/	e?tool or e?health or digital or health informat* or technolog* or telehealth* or telecon* or telemed* or

			telediag* or telemonitor* or telecom* or telecare or information communication tech* or interactive health communication or electronic shared record or electronic communic* or electronic health or online or internet* or video decision support or Videocon* or video-con* OR mobile* or m?health or app or web* or virtual
	Intervention	Dementia assessment expl/ or exp clinical assessment/ or exp clinical decision making/ or exp intervention study/	assessment or decision* or communicat* or intervention or care delivery or support or advis* or program or educat* or train* or coordinat* or integ*
